# Feasibility and acceptability of an everyday patient-centered discussion model for primary care: An exploratory, single-center study in the VA primary care setting

**DOI:** 10.1016/j.pecinn.2026.100486

**Published:** 2026-06-27

**Authors:** Frances B. Schulenberg, Sarah S. Dorin, Farah Elsiss, Joshua B. Rager, Jeremy B. Sussman, Rodney A. Hayward, Kathleen Bronson Dussán, Tanner J. Caverly

**Affiliations:** a Center for Clinical Management Research, Department of Veterans Affairs, Ann Arbor, MI, USA; b Department of Learning Health Sciences, University of Michigan School of Medicine, MI, USA; c Division of General Internal Medicine and Geriatrics, Indiana University School of Medicine, Indianapolis, IN, USA; d Center for Health Services Research, Regenstrief Institute, Indianapolis, IN, USA; e Indiana University Center for Bioethics, Indianapolis, IN, USA; f Department of Internal Medicine, University of Michigan School of Medicine, MI, USA; g VA Ann Arbor Healthcare System, Ann Arbor, MI, USA

**Keywords:** Shared decision making, Primary care, Lung cancer screening, Blood pressure treatment, Discussion model

## Abstract

**Objectives:**

Primary care visits average 15 min or less, limiting physician time for engaging in detailed shared decision-making. The ZIP approach—Zeroing in on Individualized, Patient-Centered Decisions—offers a practical path to improving decisions within time constraints. This study assesses the feasibility and acceptability of the ZIP approach in facilitating patient-centered decision-making for lung cancer screening (LCS) and blood pressure (BP) management in primary care.

**Methods:**

Multiple-methods study using audio-recorded primary care appointments and patient and physician feedback from surveys and semi-structured interviews. 23 patients who were eligible for an initial LCS (*n* = 4) or BP medication intensification (*n* = 19) and 10 primary care physicians were enrolled in a single center study in a VA medical center. Feasibility was assessed through observing duration (in minutes) for initial ZIP presentation, total conversation, completion of ZIP components, and patient-survey-based SDM measure (SDM-Q-9). Acceptability was assessed through conducting thematic analysis of patient and physician interviews.

**Results:**

ZIP discussions took less than 4 min. The median time for the initial ZIP presentation was 1.8 min (IQR) for LCS and 2 min (IQR) for BP conversations. Almost half of the encounters (43%; *n* = 9/21) had perfect ZIP fidelity scores (median = 9/10); The mean SDM-Q-9 score, reflecting patients' perceptions of the extent of SDM during the appointment, was 90.9 out of 100. Patients reported valuing personalized communication, trust, and collaborative decision-making. Physicians perceived that the SDM discussions were shorter, appreciated the tailored information and data visualizations, but suggested improvements to better align ZIP with conversational flow.

**Conclusions:**

The ZIP approach is a promising method for facilitating brief, patient-centered discussions in primary care. Its high acceptability among patients and physicians suggests strong potential for improving decision quality across diverse preventive care topics.

**Innovation:**

ZIP introduces a novel framework for integrating shared decision-making into routine care by addressing longstanding implementation barriers. Future research should examine scalability across diverse settings and evaluate sustainable approaches to integrate ZIP guidance into primary care.

## Highlights

• The ZIP approach offers an innovative, time-efficient framework for integrating shared decision-making into primary care.
• ZIP demonstrated feasibility for lung cancer screening and blood pressure management within real-world time constraints.
• Nearly half of encounters achieved perfect ZIP fidelity scores; patients rated SDM highly (SDM-Q-9 average ≈ 90.9/100).
• Patients valued personalized communication and shared control, while physicians liked the time-saving structure and visuals.
• ZIP shows promise for scaling shared decision-making across diverse preventive care topics and clinical settings.

## 1 Introduction

Routine primary care visits are often limited to 15 min, during which primary care physicians (PCPs) must navigate a complex list of patient concerns, preventive care reminders, and chronic disease management [1], [2], [3], [4], [5]. Many of these decisions are preference sensitive, where close benefit-harm tradeoffs exist, and the appropriate clinical decision depends on an individual's values and risk tolerance. Current guidelines increasingly promote using shared decision making (SDM) for “intermediate risk” patients or in cases of clinical equipoise [6], [7], [8], [9], [10]. Patient-centered decision aids have been developed to support the facilitation of SDM and are understood to increase patient engagement, accurate risk perception, and elicitation of patient preferences [11], [12]. Still, existing challenges (e.g., workflow integration, lack of adaptable delivery, time constraints, physician hesitation) [1], [2], [3], [13] limit decision aid delivery in existing SDM models for primary care (e.g., Three-Talk Model, SHARE) [2], [14]. Additionally, current SDM models, which typically require 5–10 min of initial presentation, are impractical because PCPs usually have less than 5 min for any preventive care discussion [4], [5], [15], [16]. On average, SDM conversations take 11.6 min to complete, while preventive care topics realistically have fewer than 3 min allotted for a single decision, highlighting time as a major systemic barrier of implementing SDM in routine practice [17], [18], [19]. Therefore, we propose a practical alternative: a novel and ‘everyday’ version of SDM called the ‘ZIP’ approach – ‘Zeroing in on Individualized, Patient-Centered Decisions’ – to facilitate patient-centered discussions within primary care time-constraints [4], [22]. ZIP is a compromise approach designed to balance feasibility with the physician's dual role of supporting patient autonomy and acting as a gentle health advocate [4].

The ZIP approach streamlines patient-centered discussions into three essential steps: (1) making a personalized recommendation, (2) briefly presenting qualitative information on key tradeoffs, and (3) conveying full support for a patient's decisional autonomy and desire for more information. Although initially called the “everyday SDM” approach in earlier work, the approach was renamed based on feedback from PCPs that “SDM” implies “not doable” within the constraints of primary care settings [1]. Previous research has shown the ZIP approach's acceptability through focus groups with patients [5]. This study evaluates the acceptability and feasibility of the ZIP approach during actual primary care visits, focusing on two common primary care decisions where a brief, effective, patient-centered approach is necessary—lung cancer screening (LCS) and blood pressure (BP) management [4].

## 2 Methods

### 2.1 Overview

Full study methods have been published [22]. We used multiple-methods study design, gathering quantitative and qualitative patient and physician data to assess the feasibility and acceptability of the ZIP approach [20], [21], [22]. This study follows Standards for Reporting Qualitative Research (SRQR) (Appendix H) [38]. This study focused on personalizing two decisions through ZIP: (1) starting annual low-dose computed tomography (LDCT) LCS for patients meeting current US Preventive Services Task Force (USPSTF) eligibility criteria and (2) intensification of BP treatment (i.e., prescribing a BP medication for the first time, adding an additional BP medication to the current regimen, or increasing the dose of current medications) for primary prevention of atherosclerotic cardiovascular disease (ASCVD). LCS and BP interventions were selected for this study because they are common primary care decision models where prediction models exist and individualized information is known to be impactful. These two interventions were used as a starting point in this pilot to examine how ZIP, using more individualized risk/benefit information, could support personalized SDM discussions in primary care settings. Because low-benefit patients were excluded from this pilot, physicians did not encounter situations in which discouraging LCS or BP treatment was necessary.

Study activities included a physician education session, audio-recorded medical encounter, and post-appointment surveys and interviews with patients and physicians. As part of the ZIP approach, PCPs were given an encounter-based paper decision support tool to guide their recommendations with recruited patients. This approach was measured against feasibility criteria (i.e., the ability to implement all components of the ZIP approach within primary care appointments) and acceptability criteria (i.e., the extent to which patients and physicians found the approach appropriate based on their preferences and experiences).

### 2.2 Ethical considerations:

Written informed consent was obtained from patients and PCPs prior to research activities. IRB approval was obtained 02/09/2023 (Reference #1725106). Patient privacy, human rights, and personal information regulations were followed. Patient data was de-identified and can be made available upon request.

### 2.3 PCP recruitment and education

We recruited 10 PCPs with at least 50% of their time devoted to ambulatory care from a single VA Medical Center (VAMC). A total of 21 PCPs were approached, until 10 PCPs agreed to participate (participation rate = 47%). Purposeful sampling was conducted to ensure representation of genders, ages, and experience level. The research team held one-on-one training sessions with each participating physician. All 10 enrolled PCPs completed a full 1-h training session, which included a 25-min narrated slide deck followed by time for questions and practice. The slide deck covered BP and LCS preference-sensitive decision-making, specific language for delivering brief, personalized recommendations, and how the ZIP approach addresses challenges in time-constrained primary care (Appendix C). Materials were developed based on prior study content. If time remained in the session, physicians participated in a patient scenario activity where they could practice the ZIP Approach with hypothetical patient scenarios across diverse benefit and patient preference styles. These scenarios were also emailed to physicians prior to seeing their study patients.

### 2.4 Patient recruitment

Eligible patients of the recruited PCPs were identified through medical chart review and contacted for participation. Across the 96 eligible patients contacted, 23 agreed to participate, yielding a 24% participation rate. We sought to recruit 2–3 patients from each PCP, with a target of at least 1 LCS and at least 1 BP patient. Full eligibility criteria have been previously published [22]. Patients were mailed a recruitment letter and consent form, informing them of the study and inviting participation. A study team member (FS) called each patient 2–3 days later to assess their interest in participating, where up to 3 attempts to contact were made. Patients were enrolled in the study between October 31st, 2024, and April 24th, 2025.

### 2.5 ZIP decision support tool

**Fig. 1:**
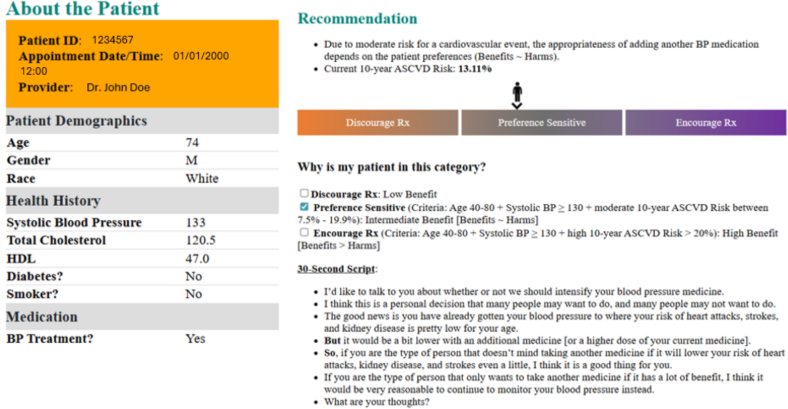
Example Blood Pressure Net Benefit Spectrum and Discussion Points. *ASCVD = Atherosclerotic Cardiovascular Disease.

**Fig. 2:**
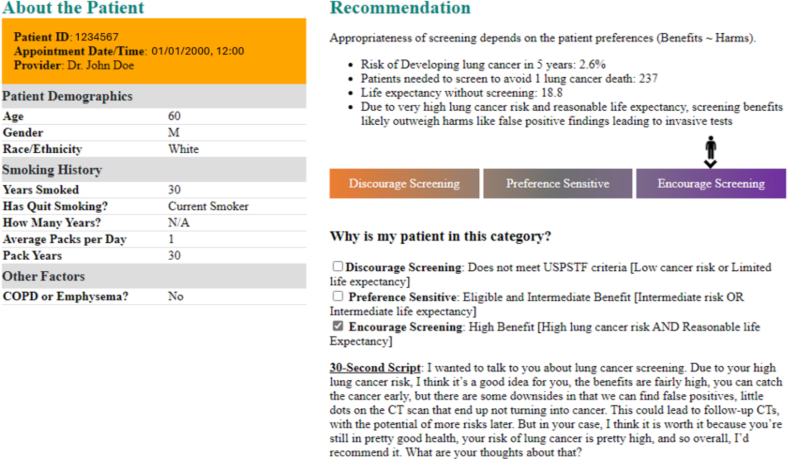
Example Lung Cancer Net Benefit Spectrum and Discussion Points

Prior to each study visit, PCPs received a paper-based decision support tool, modeled on well-validated prediction models and a previously developed web-based tool [9], [22], [23], [24], [25], [26], [27], [28], [29], [30]. This tool presented individualized predictions for LCS or BP treatment decisions, along with a visual “net benefit spectrum” categorizing patients into either an “encourage zone” or “preference-sensitive zone” [28], [29], [30], [31]. The “encourage zone” indicates situations where the chance of net benefit is high and where a more clear-cut recommendation is appropriate. The “preference-sensitive zone” indicates a closer match between benefits and harms, highlighting the importance of evaluating and acting on patient preferences. Risk factors considered for LCS included age, smoking duration, smoking intensity, quit status, and COPD/emphysema diagnosis [28], [29], [30]. For BP management, risk factors included age, HDL, total cholesterol, systolic blood pressure, smoking status, and diabetes status [31], [32]. The paper decision tool included example discussion points to support delivery (Table 1; Fig. 1, Fig. 2). Overall, the ZIP Decision Support Tool is designed to help PCPs quickly visualize patient risk and net benefit, to support gentle health advocacy in zones of clear-cut higher net benefit and patient autonomy for gray-area decisions. The ZIP tool differs meaningfully from other decision aids and systems by integrating both prediction-based stratification and graded communication guidance into a single, point-of-care tool.

**Table 1:** Zeroing in on the Individualized, Patient-Centered Decisions approach: Example recommendation scripts.

	Encourage	Preference Sensitive
Blood Pressure	• “I'm going to recommend we intensify your BP medication. Your risk of heart attacks and strokes has gone up; it is normal for your risk to go up with age.” • “But we have found that additional medication can help because it decreases your blood pressure over the course of the whole day. This helps protect your brain, kidneys, and heart.” • “What your blood pressure reading is in the office today is not what's most important. What's important is your blood pressure when you are around and active over the course of the day.” • “It's one additional medication [or a higher dose of a medication]. If you have any side effects, which are rare, we can stop it. So, I strongly recommend us trying that.” • “What are your thoughts?”	• “I'd like to talk to you about whether or not we should intensify your blood pressure medicine.” • “I think this is a personal decision that many people may want to do, and many people may not want to do.” • “The good news is you have already gotten your blood pressure to where your risk of heart attacks, strokes, and kidney disease is pretty low for your age.” • “But it would be a bit lower with an additional medicine [or a higher dose of your current medicine].” • “So, if you are the type of person that doesn't mind taking another medicine if it will lower your risk of heart attacks, kidney disease, and strokes even a little, I think it is a good thing for you.” • “If you are the type of person that only wants to take another medicine if it has a lot of benefit, I think it would be very reasonable to continue to monitor your blood pressure instead.” • “What are your thoughts?”
Lung Cancer Screening	• “I wanted to talk to you about lung cancer screening. Due to your high lung cancer risk, I think it's a good idea for you.” • “The benefits are fairly high; you can catch the cancer early. There are also some downsides in that we can find false positives, little dots on the CT scan that end up not turning into cancer. This could lead to follow-up CTs.” • “But in your case, I think it is worth it because you're still in pretty good health and your risk of lung cancer is pretty high. So overall, I'd recommend it.” • “What are your thoughts about that?”	• “I wanted to talk to you about lung cancer screening. It's something to consider; for you it's not a clear decision. There are some pros and cons. The big pro is a chance of catching the cancer early and curing it.” • “But there are also cons like a risk of false positives which are little dots on the CT scan that end up not turning into cancer. This could lead to follow-up CTs.” • “The good news is you're not at super-high risk; it's a little bit of benefit with a little bit of downside. So, it's really a personal choice between how you feel about those pros and cons.” • “What are your thoughts?”

### 2.6 Net benefit zone classification

Patients were placed in the preference-sensitive or encourage zone based on individualized multivariable prediction: days of life gained for LCS or 10-year atherosclerotic cardiovascular disease risk (ASCVD) for BP treatment intensification. Based on prior work and recommendations in national guidelines [[24], [33], [34]], LCS patients were placed in the preference-sensitive zone if predicted life-gain was <16.2 days, and in the encourage zone if life-gain was ≥16.2 days [24], [25]. For BP treatment decisions, patients were categorized as preference-sensitive with an ASCVD risk of 7.5–19.9%, and as encourage if risk was ≥20%, consistent with national guideline thresholds [26], [27], [28]. Full details are previously published [22].

### 2.7 Intervention

Physicians were contacted via Microsoft Teams 1–2 days before their recruited patient's appointment. Patients arrived 30 min early to confirm participation, review study activities, and sign informed consent. Patients were taught how to use a digital voice recorder (DVR) and told to give the decision aid to the nurse prior to seeing their PCP. The DVR was turned on by the physician and patient for the entire duration of the appointment. Physicians followed the ZIP tool to present their recommendations. After the appointment, patients completed a survey that included demographic information, decision-making, and medical trust, measured using three Likert-scale items assessing trust in the PCP, local VA system, and national VA system. (Appendix E). Patients then completed a 10–20-min interview about their visit (Table 2). The interviewer (FBS) was a research assistant with training in qualitative interviewing and no clinical role, which minimized power dynamics during patient interviews. All interviews were audio recorded. Each patient received a $25 gift card as remuneration for their time.

**Table 2:** Key Findings from Semi-Structured Interviews.

Patient perspective on the ‘Zeroing in on Individualized, Patient-Centered Decisions’ (ZIP) approach
• Increased feelings of respect and involvement (13/22 total patient participants); (2/4 LCS^a^); (11/18 BP^b^) • Appreciated its similarity to previous conversations and decision-making process with their doctor (12/22); (3/4 LCS); (9/18 BP) • Liked receiving information and a recommendation followed by open discussion (14/22) (3/4 LCS); (11/18 BP) • No negative comments or recommended changes to the ZIP approach (22/22); (4/4 LCS); (18/18 BP)
Physician perspective on the ZIP approach
• Aligned with their clinical practice (5/10 physicians) • Deemed it a time-saver and a more feasible decision-making approach for a busy clinic (3/10) • Endorsed it as a helpful way to formulate quality recommendations for individual patients (7/10) • Appreciated the patient-friendly language used in the example discussion points (2/10)
Physician suggestions for optimizing the ZIP approach
⦁ Provide more guidance on how to flexibly incorporate example ZIP discussion points into conversational flow (8/10 physicians) ⦁ Make sure that all patient information is up-to-date, even if the medical record is not (4/10) ⦁ Strongly endorsed efforts to incorporate ZIP tools into the EHR (2/10) ⦁ Expressed a strong desire that ZIP tools be developed for additional common primary care decisions (9/10)

a Lung Cancer Screening.

b Blood Pressure.

After completing 2–3 study visits, physicians were contacted to schedule a 30-min interview and demographic survey. They provided feedback on the decision aid and its feasibility during appointments (Table 2). As VA employees, physicians were not eligible for compensation.

### 2.8 Feasibility (ability to implement ZIP approach within time-constraints)

All quantitative data (timing, ZIP Scale scores, and SDM-Q-9 scores) were summarized descriptively. Because this was a pilot feasibility study with a small sample size, no statistical hypothesis testing or inferential analyses were conducted.

*Timing and Duration*. Feasibility was assessed by timing the physician's initial ZIP presentation (introduction of topic, presentation of patient's risk, and recommendation) and the full conversation (patient input, elicitation of patient preferences, clarification of questions, final decision) through audio-recorded timestamps.

*ZIP Scale*. Fidelity was evaluated with a 5-item ‘ZIP Scale,’ which the team developed for this study because no validated instrument previously existed for this approach. The ZIP scale scores the physician's ability to deliver personalized recommendations (2 items), present qualitative information on pros and cons (1 item), and respect patient autonomy (2 items) [22]. Each item is rated 0 to 2 (0 = not attempted; 1 = partial; 2 = completed), with a maximum total summed score of 10 points. Full score distributions (Table 3, Appendix B) and development details (Appendix D) are available.

**Table 3:** ‘Zeroing in on Individualized, Patient-Centered Decisions’ (ZIP) Score, Shared Decision Making Questionnaire 9 (SDM-Q-9) Score, Initial Presentation, and Total Conversation by Recommendation Zone and Topic.

Patient ID	Topic	Risk Group	SDM-Q-9 Score (X/100)	ZIP Score (X/10)	Initial Presentation (Minutes: Seconds)	Total Conversation (Minutes: Seconds)
1009	LCS^a^	E^b^	100	10	1:14	2:21
1011	LCS	E	100	10	1:38	5:00
1017	LCS	E	83.3	10	2:22	2:57
1023	LCS	E	100	10	3:40	10:40
2002	BP^c^	E	87.04	9	1:25	2:14
2006	BP	E	96.3	10	1:32	5:20
2009	BP	E	92.6	⁎	⁎	⁎
2050	BP	E	81.5	9	NA	NA
2065	BP	E	⁎⁎	7	1:49	2:09
2069	BP	E	81.5	10	3:40	4:10
2084	BP	E	100	9	1:49	3:44
2095	BP	E	90.7	9	4:10	6:45
2133	BP	E	96.3	⁎	⁎	⁎
2018	BP	PS^d^	100	10	3:23	4:03
2043	BP	PS	83.3	7	1:46	3:23
2056	BP	PS	100	8	1:20	1:20
2058	BP	PS	100	8	1:20	3:13
2080	BP	PS	53.7	9	3:07	4:00
2081	BP	PS	⁎⁎⁎	9	2:25	2:52
2104	BP	PS	100	⁎	⁎	⁎
2111	BP	PS	100	10	2:05	6:45
2116	BP	PS	96.3	10	1:15	3:35
2128	BP	PS	100	6	0:50	1:00

a = Lung Cancer Screening.

b = Encourage.

c = Blood Pressure.

d = Preference-Sensitive.

⁎ No Medical Encounter (No ZIP score, Initial Presentation, Total Conversation).

⁎⁎ No Interview/Survey (No SDM-Q-9 Score).

⁎⁎⁎ Missing Survey Answers (No SDM-Q-9 Score).

Two study team members (FBS, FE) independently scored transcripts, resolving any discrepancies through discussion, and if needed, full-team review (FBS, FE, SD, TJC). Inter-rater agreement was moderate (Pearson Correlation = 0.64) and weaker for lower ZIP scores (5–8) than higher ZIP scores (9–10). Item 3 (offering pros/cons) had the highest disagreement (*r* = 0.33), while Item 1 (offering a personalized recommendation), showed perfect alignment (*r* = 1).

*SDM-Q-9.* Patient perception of SDM was measured with the SDM-Questionnaire-9 (SDM-Q-9) [35]. This 9-item questionnaire (with 0 points indicating minimal SDM support and 54 points indicating maximum support) [35] was evaluated as part of the patient survey and converted to a 0–100 scale for easier comparison.

### 2.9 Acceptability

Patients and physicians participated in semi-structured interviews to assess ZIP's perceived acceptability (Appendix E and F). Patient interviews focused on the appointment experience and perception of benefits and harms of LCS or BP treatment. Physician interviews discussed ZIP implementation, effectiveness, and timing, as well as their perceptions of the benefits and harms of LCS and BP medications.

All interviews were audio recorded, de-identified, and transcribed. The study team conducted rapid thematic analysis using deductive coding based on interview questions. Quotations presented in the results section are illustrative of these coded domains. All direct quotes come from post-visit patient and physician interviews; full transcripts of preference-sensitive physician ZIP presentations can be found in Appendix G. Observational data were used only for timing, fidelity scoring, and descriptive summaries. Acceptability was assessed based on participants' descriptions of whether the ZIP approach felt appropriate, understandable, and useful in the context of the clinical encounter. Each transcript was independently reviewed and coded (FBS, FE), with unresolved discrepancies discussed in team meetings. One patient completed the medical encounter but missed the survey and interview due to a conflict; three encounters were incomplete—two from technical issues and one patient opting out of recording. This data is reported as missing. No additional information about patient decision making beyond the medical encounter, interview, and survey were available.

## 3 Results

### 3.1 Participant characteristics

A total of 10 PCPs were enrolled in the study, with half female (*n* = 5, 50%), 40% (*n* = 4) Asian, and 40% (n = 4) White. Complete PCC demographics are available in Table 4. Ninety-six eligible patients were identified, 23 for first-time LCS and 73 for BP medication intensification. Four patients in the LCS group and nineteen in the BP group participated. Full details are listed in the recruitment consort diagram (Appendix A) [22]. Most participating patients (*n* = 21 of 23) were male and White, averaging 69 years old. Ten patients (43%) had been with their physicians for over 5 years, and most (n = 21/23) expressed strong trust in their doctor. Based on risk factors, all LCS patients (*n* = 4) were in the encourage zone; BP patients were split (10 preference-sensitive, 9 encourage). Complete patient demographics are in Table 5.

**Table 4:** Physician Demographics.

Total	10
Gender, n (%)	
Male	5 (50)
Female	5 (50)
Race, n (%)	
White	4 (40)
African American	0 (0)
Asian	4 (40)
Missing	2 (20)
Hispanic or Latino	
Yes	1 (10)
No	9 (90)
How long since completed clinical training, n (%)	
<5 years	2 (20)
5–10 years ago	2 (20)
11–19 years ago	2 (20)
20+ years ago	4 (40)
How long practicing at the VA	
<5 years	3 (30)
5–10 years	1 (10)
11–19 years	6 (60)
20+ years	0 (0)
Average days per week working in Primary Care	
1–2 days a week	3 (30)
3–4 days a week	7 (70)
5+ days a week	0 (0)

**Table 5:** Patient Demographics.

	Total	LCS	BP
Total	23	4	19
Sex, n (%)			
Male	21 (91)	4 (100)	17 (89)
Female	1 (4)	0 (0)	1 (5)
Missing	1 (4)	0 (0)	1 (5)
Race and Ethnicity, n (%)			
White	21 (91)	4 (100)	17 (89)
African American	1 (4)	0 (0)	1 (5)
Missing	1 (4)	0 (0)	1 (5)
Hispanic or Latino			
Yes	1 (4)	0 (0)	1 (5)
No	22 (95)	4 (100)	18 (95)
Age, mean (sd)	69 (6.6)	66 (6.5)	70 (6.6)
Education, n (%)			
High school	4 (17)	0 (0)	4 (21)
Some college/Trade School	10 (43)	3 (75)	7 (37)
Associate's Degree	4 (17)	0 (0)	4 (21)
Bachelor's Degree	2 (9)	1 (25)	1 (5)
Master's Degree or more	2 (9)	0 (0)	2 (10)
Missing	1 (4)	0 (0)	1 (5)
Benefit Category, n (%)			
Preference Sensitive	10 (43)	0 (0)	10 (53)
Encourage	13 (57)	4 (100)	9 (47)
Patient Provider Relationship, n (%)			
New Relationship (<2 years)	3 (13)	0 (0)	3 (16)
2–5 years	7 (30)	2 (50)	5 (26)
6–10 years	3 (13)	1 (25)	2 (10)
11–15 years	4 (17)	0 (0)	4 (21)
>15 years	3 (13)	1 (25)	2 (10)
Missing	3 (13)	0 (0)	3 (16)
Complete Trust in Doctor			
Somewhat Agree	1 (4)	0 (0)	1 (5)
Strongly Agree	21 (91)	4 (100)	17 (89)
Missing	1 (4)	0 (0)	1 (5)

### 3.2 Feasibility – Timing, ZIP Fidelity, SDM-Q-9

ZIP presentations averaged 1.8 min; 1.8 min for BP (range 0:50–4:10), 2 min for LCS (range 1:14–3:40), 1.7 min for preference-sensitive conversations (range 0:50–3:23), and 1.8 min for encourage conversations (range 1:14–4:10). Full conversations averaged 3.6 min: 3.6 min for BP (range 1:00–6:45); 3.9 min for LCS (range 2:21–10:40); 3.4 min for preference-sensitive conversations (range 1:00–6:45); and 3.95 min for encourage conversations (range 2:09–10:40). ZIP presentations and total conversations across recommendation zones and topics took similar amounts of time.

Using the 5-item ZIP scale, we found that nearly half of the 20 audio-recorded appointments (*n* = 9) achieved a perfect score (4/4 LCS appointments and 5/16 BP appointments); others scoring 6–9 points. For preference-sensitive zone patients (n = 9), scores were evenly distributed between 6 and 10, whereas in the encourage zone (*n* = 11), most scored 9 or 10. Overall, there were no stark differences or patterns in ZIP scoring across BP vs. LCS and preference-sensitive vs. encourage zone conversations although the study was not powered to detect differences. Item 3 (discussing pros/cons) was least complete (Appendix B), with 30% of appointments scoring partial points and 10% scoring no attempt.

The SDM-Q-9 scale, although not a direct measure of fidelity to the ZIP approach, indicates whether patients perceived key components of SDM. Of 21 patients surveyed, 10 (48%) reported a perfect SDM-Q-9 of 100, and 5 (24%) scored in the 90s, with the remaining 6 patients (28%) scoring between 50 and 80 with no clear patterns or differences across BP vs. LCS and preference-sensitive vs. encourage zone conversations (Table 3).

### 3.3 Acceptability - Interviews

Summarized high-level findings from semi-structured physician and patient interviews can be found in Table B.

*Patient Acceptability*. When prompted to reflect on the ZIP conversation during post-visit interviews, many patients reported positive interactions with their physicians (*n* = 19/22; 86%) and a feeling of respect and involvement in their healthcare decision-making (*n* = 13/22; 59%). One patient noted, “[my] voice was heard” (ID2116).

An important consideration in understanding the acceptability and generalizability of this approach was that many participants described a pre-existing foundation of trust with their PCP (*n* = 18/22; 81.8%). One patient who had seen their physician for 17 years, emphasized, “She's prepared because she knows me. So, when I come in to see her, she doesn't have to look anything up, we don't have to play a guessing game.” This level of trust engendered confidence their “doctor already has a plan” and is “not wasting [their] time.” (ID2116). One patient reflected that when “you can trust someone that you understand… it's not a cold clinical evaluation. There's a personal touch to that and that makes it seem a little bit more reasonable… you're more receptive to what they have to say… they're hearing what you're saying” (ID2111). Established trust allowed patients to participate in decision-making, feeling heard and confident.

In this context of trust and established relationships, half of patients (12/22; 50%) noted that the ZIP approach was familiar and similar to prior valued conversations. As one patient mentioned, “[it's] what I have gotten and what I expected.” (ID2006). Others described their interactions as typical and casual, stating they “just chatted” (ID2128) and that it's “just her general technique” (ID2081). The ZIP presentation was not viewed as a deviation from the standard conversational dynamic, but as fitting into their PCP's typical approach.

Rather than perceiving the physician's ZIP presentation as paternalistic, patients appreciated communication tailored to their medical history, personality traits (e.g., ‘stubbornness’ (ID1009), or specific mental health concerns (ID1017). Patients appreciated the clarity of their physician's ZIP presentation, which reduced ambiguity and anxiety regarding medical decision making.

Finally, patients (20/22; 90.9%) felt the ZIP approach aligned with their explicit desire for shared control in decision-making and clear recommendations from their PCP. As indicated in the survey, nearly all patients (*n* = 20/22/; 90.9%) preferred to share the responsibility with their physicians or make the final decision after considering a PCP's initial recommendation. In interviews, patients emphasized this collaboration, with one describing it as “an open discussion” to ask, “is this really the best thing for me?” (ID1011). Another patient noted, “I've had no medical training, I just know how I want to feel, I know what my goals are… and he knows what those things are, and so we have a common goal; mobility and quality of life, so, yeah, we're on the same wavelength” (ID2002). Overall, patients mentioned the importance of being active patients (4/22; 18%), with one reflecting, “being a good patient is asking the right questions, listening to your doctor… and if you don't understand, you ask again” (ID2018). Notably, no negative aspects of the ZIP approach were mentioned, reinforcing the overall positive reception.

*Physician Acceptability:* Physicians also expressed positive feelings when describing their interactions with the ZIP approach (*n* = 5/10). One physician shared, “I feel like I do shared decision making a lot… but I think it was helpful to really have it spelled out and learn from the script I was given” (ID102). The ZIP approach was perceived as a time-saver, particularly in preference-sensitive cases, allowing physicians to quickly transition to other tasks in the instance when a patient expressed disinterest in the intervention (*n* = 3/10). Though there were no LCS patients in the preference-sensitive zone, physicians used varying approaches to convey their patient's intermediate level of benefit for BP patients. One physician spelled it out: “If you're the type of person that doesn't mind taking another medication … I think (it's) a good thing for you. But if you're the type of person that only wants to take another medication if it has tremendous benefit, I think it'd be reasonable to continue to monitor you, continue to make lifestyle choices.” (ID108). Full transcripts of preference-sensitive ZIP presentations can be found in Appendix G.

*Physician Acceptability – Decision-Support Tool:* Physicians found that risk information and data visualization enhanced their understanding of patient's risk, strengthened their recommendations, and provided a sound basis for justifying medication intensification or screening. As one physician stated, the data visualizations provide “evidence for why we were recommending either certain medication(s) or screening(s)” (ID102) and another reflected, “I think in one situation, I may not [have] even thought… about adding a medication, so it kind of reminded me to do it” (ID104). Others noted that the example ZIP presentation helped them understand patient risks and that employing the ZIP presentation style helped patients understand their risk as well (6/10; 60%), even motivating one high-benefit patient to reconsider a previously declined screening decision. The tool served as a reminder to patients and physicians alike, “Maybe I should nudge him a little bit on this, even though we've had this conversation” (ID110). The patient-friendly language of the example ZIP discussion points was mentioned by 2/10 PCPS, as it “uses a lot of layman terms for people that may not be as familiar with the medical terminology” (ID102). The ZIP approach also seemed to positively influence attitudes towards SDM. One physician reflected on their medical school experiences, where SDM was often portrayed as an overly rigid model that left physicians feeling they could no longer comfortably make recommendations. In contrast, they reflected that the ZIP approach was described as helping “bring more balance,” offering a structure that supports patient autonomy without “putting it all on the patient” (ID107).

Most physicians identified challenges with the decision tool and offered constructive suggestions for improvements (*n* = 8/10). One challenge was the tool's language and structure, which inadvertently led one physician to see the discussion points as a script to be followed rigidly rather than, as intended, as sample discussion points to loosely guide the brief ZIP conversation. This played out in two appointments (*n* = 2/10) where the tool limited patient interaction and led to patient interruptions. One physician reflected they could only get “the first two bullet points out” before being interrupted and never being “able to get through the whole script” (ID105). Physicians recommended clarifying the intention of loosely guiding the conversation and offering more guidance on how to flexibly incorporate the information into conversational flow (8/10; 80%). Physicians also discussed other logistical challenges like the need to consider multiple BP treatment options during the ZIP presentation (e.g., starting a new medication, intensifying the current dose, collecting additional BP data, or referring to a specialist) – which complicates the ZIP discussion – and up-to-date medical information in order to accurately inform ZIP (e.g., full current antihypertensive regimen) (4/10; 40%). However, physicians expressed enthusiasm for integrating the ZIP approach broadly into their practice due to its versatility and usefulness. PCPs discussed the benefits of incorporating ZIP decision tools into EHR (2/10; 20%) or distributing it via Microsoft Teams prior to the medical encounter (3/10; 30%). Physicians recognized the ZIP approach's potential in diverse preventive health areas, including diabetes care, vaccinations, medication management, pain relief, and obesity treatment. Nearly all physicians (9/10) expressed a desire for ZIP tools to be developed for these types of common primary care decisions. Physicians emphasized that the ZIP approach could be especially useful in situations where preventive care topics do not have a universally set standard for recommendation across patients (e.g., colon cancer screening over age 75), for conversations focusing on risks and benefits (e.g., vaccinations), and when there are multiple or alternative options tto consider (e.g., decisions to start narcotics). Overall, the ZIP approach was viewed as a useful tool that has the potential to enhance care quality across diverse clinical topics.

## 4 Discussion and Conclusion

### 4.1 Discussion

Our exploratory findings from a single VA Medical Center suggest that simplifying SDM conversations using the ZIP approach is acceptable and likely to be more feasible than traditional SDM approaches when time is limited. Patients responded positively, noting that the ZIP approach promoted patient understanding, enhanced rapport building, and maintained confidence in their PCP's decision-making. Primary care physicians also supported the ZIP approach and perceived the approach helped them save time when facilitating patient-centered discussions about risk.

Prior research shows the ZIP approach is acceptable to patients, [5], [36], [37] and this exploratory study is the first to evaluate its real-world feasibility and acceptability. These findings are broadly consistent with prior evidence that ZIP-based decision support tools increase LCS uptake among high benefit patients [2], [9], [11], [32]. Despite the small LCS sample (*n* = 4) and exclusively ‘encourage zone’ patients, our findings suggest ZIP may be particularly straightforward and acceptable for high-benefit LCS discussions. Also, SDM-Q-9 and ZIP scores were nearly perfect across the 4 LCS conversations, in contrast to more variability observed across the 19 BP conversations. This study was designed as a small pilot study focused on feasibility and acceptability rather than statistical comparison differences across groups, allowing the team to analyze close observation of the ZIP approach in clinical encounters. These findings support the need for larger comparative effectiveness studies examining ZIP versus other SDM models, such as SHARE or the Three-Talk Model [2], [14].

This pilot was not designed to conduct direct comparisons with usual care; thus, future research will support the evaluation of ZIP's effectiveness relative to existing practices in LCS decision-making. Currently, usual care conversations for LCS are extremely brief, omit key LDCT harms, and do not often include patient-education materials [39]. And across the board, core SDM elements appear only in a minority of routine visits and conversations skip explicit discussion of options and harm-benefit information [40], [41], [42]. Especially as compared to existing practice, we see the ZIP approach as a step in the right direction.

Our study was limited by a single center design within the VA primary care setting. We had a small physician sample size (*n* = 10), and a small patient sample size (*n* = 23) with a small LCS cohort (*n* = 4).The patient response rate was relatively low (24%); Patients who participated potentially had higher levels of pre-existing trust and engagement, which could have introduced a bias into ZIP's acceptability. The enrolled patient population was predominantly white males. Patients who agreed to participate were likely to have an existing positive relationship with their physicians. This form of selection bias limits its generalizability, as trust is a precursor for patient involvement [43] and patients who have trust in their physician are more likely to accept their recommendations for care. In the context of high-trust patient-physician relationships, findings should be interpreted as acceptability and feasibility of the ZIP approach within the context of ongoing physician–patient relationships, rather than as evidence of effects attributable solely to the ZIP tool or training. Nevertheless, this study provides the first exploratory evidence of the ZIP approach. Future research should include multiple centers with more diverse populations, evaluate ZIP's effectiveness across other preference-sensitive decisions, and further examine how patients interpret qualitative terminology during ZIP discussions.

### 4.2 Innovation

ZIP represents an innovative solution to longstanding barriers in implementing SDM in primary care settings. Unlike traditional SDM models that require 5–10 min for the initial SDM presentation, ZIP enables meaningful, patient-centered discussions in under 4 min [2]. It's structured and flexible design is easy to learn, with half of physicians (*n* = 10) achieving perfect ZIP fidelity scores after a single 30–60-min training session.

Patients rated ZIP highly (SDM-Q-9), highlighting its ability to meet core SDM objectives [4], [36]. Importantly, patients perceived the ZIP approach to be aligned with their physician's existing communication style, supporting easier adoption without disrupting valued communication styles and relationships. Physicians suggested improvements, such as bullet-point discussion prompts and succinct guidance, to improve flexible integration into future primary care workflows.

ZIP is a novel approach to advancing SDM goals in time-constrained primary care settings. Future research should evaluate ZIP's scalability across diverse care settings and explore innovative strategies for embedding ZIP guidance into workflows to enhance accessibility and sustainability. Implementing ZIP tools at scale would require automated risk-calculation tools and clinical reminders to be directly integrated into EHRs. Without this integration, the burden of preparing tailored risk information would impede ZIP's clinical feasibility.

## 5 Conclusion

The ZIP approach, including the discussion points and visuals, was found to be acceptable and useful for patient-centered discussions during primary care visits. Scaling ZIP to support SDM across diverse populations and for a broader range of everyday decisions in primary care represents a promising new area of investigation for achieving the goals of shared decision making and advancing patient-centered care.

## Declaration of Generative AI and AI-assisted technologies

Microsoft Co-Pilot was used to assist with grammar, clarity, and reduction of word count during the preparation of this manuscript for publication. After using this tool, the paper was reviewed and revised. No AI tool was used to analyze or interpret data.

## CRediT authorship contribution statement

**Frances B. Schulenberg:** Writing – review & editing, Writing – original draft, Visualization, Validation, Resources, Project administration, Methodology, Investigation, Formal analysis. **Sarah S. Dorin:** Writing – review & editing, Writing – original draft, Visualization, Validation, Resources, Project administration, Methodology, Investigation, Formal analysis. **Farah Elsiss:** Writing – review & editing, Validation, Investigation, Formal analysis. **Joshua B. Rager:** Writing – review & editing, Validation, Investigation. **Jeremy B. Sussman:** Writing – review & editing, Validation, Resources, Investigation, Data curation. **Rodney A. Hayward:** Writing – review & editing, Validation, Software, Investigation, Data curation. **Kathleen Bronson Dussán:** Writing – review & editing, Validation, Investigation. **Tanner J. Caverly:** Writing – review & editing, Writing – original draft, Validation, Supervision, Software, Resources, Methodology, Investigation, Funding acquisition, Formal analysis, Data curation, Conceptualization.

## Funding information

This work was supported by the 10.13039/100000738VA HSR&D (Grant Number SFT 23–301). This paper was presented at the Society for Medical Decision Making 47th Annual Meeting for an oral presentation (June 17th, 2025) and poster presentation (June 18th, 2025) in Ann Arbor, Michigan.

## Declaration of competing interest

Dr. Tanner J. Caverly reports holding an Apache 2.0 license for DecisionPrecision, a freely available, open-source tool to enable personalized shared decision-making for lung cancer screening. He does not receive any remuneration of any kind related to this tool. The remaining authors have no funding or conflicts of interest to disclose.

Ethics Approval and Consent:

Informed consent was obtained from all participants prior to any research activities. This study received Institutional Review Board initial approval on February 9th, 2023 (Reference Number: 1725106).

## Figures and Tables

**Figure:**
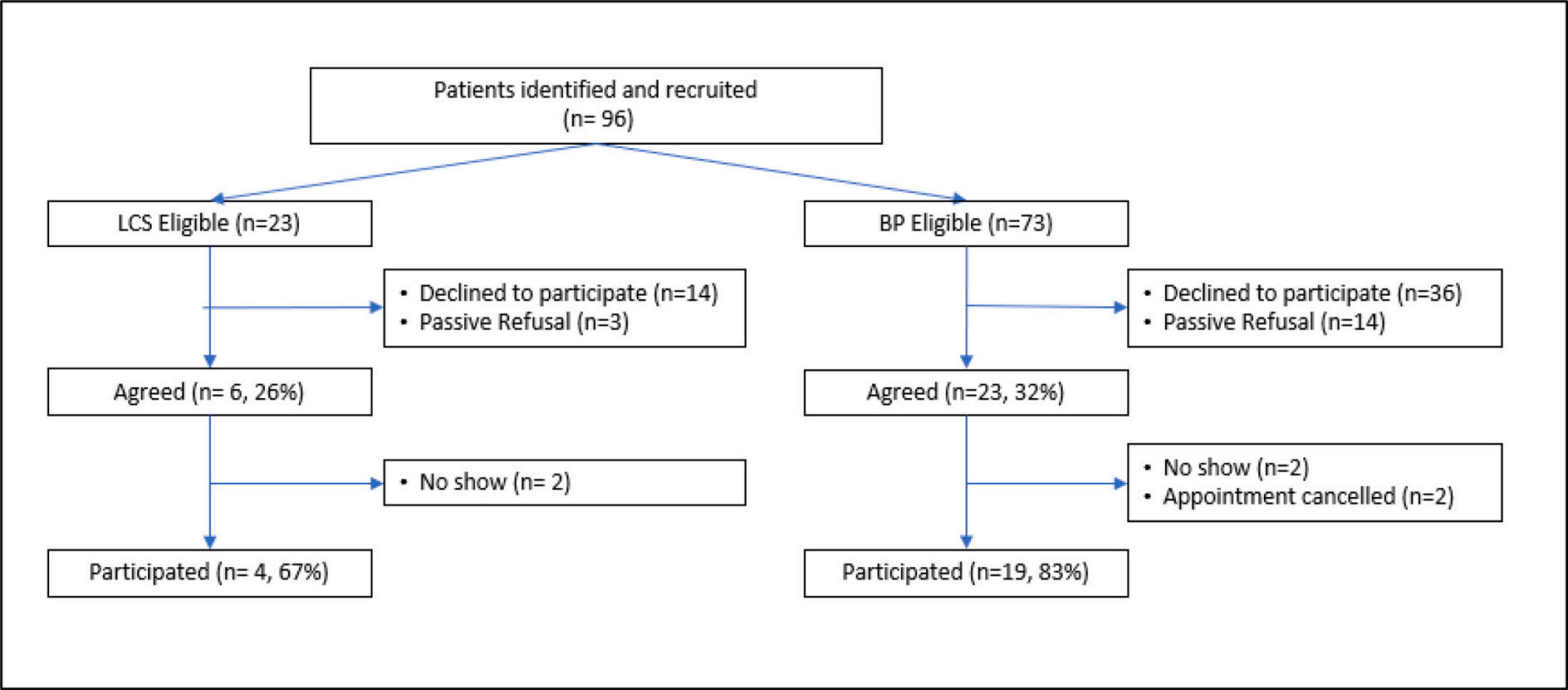


**Table:** 

Patient ID*	Topic	Risk Category	Item 1: Personalized Recommendation to risk factors?	Item 2 A: Personalized to preference-sensitive/encourage zone?	Item 3: Presentation of qualitative pros/cons?	Item 4: Support patient requests for more information?	Item 5: Support patient as final decision maker?	Composite Score
1009	LCS^a^	E^c^	2	2	2	2	2	10
1011	LCS	E	2	2	2	2	2	10
1017	LCS	E	2	2	2	2	2	10
1023	LCS	E	2	2	2	2	2	10
								
2002	BP^b^	E	2	1	2	2	2	9
2006	BP	E	2	2	2	2	2	10
2050	BP	E	2	1	2	2	2	9
2065	BP	E	2	2	1	2	0	7
2069	BP	E	2	2	2	2	2	10
2084	BP	E	2	2	2	2	1	9
2095	BP	E	2	2	1	2	2	9
								
2018	BP	PS^d^	2	2	2	2	2	10
2043	BP	PS	2	1	0	2	2	7
2056	BP	PS	2	1	1	2	2	8
2058	BP	PS	2	2	0	2	2	8
2080	BP	PS	2	2	1	2	2	9
2081	BP	PS	2	2	1	2	2	9
2111	BP	PS	2	2	2	2	2	10
2116	BP	PS	2	2	2	2	2	10
2128	BP	PS	2	0	1	2	1	6

**Figure:**
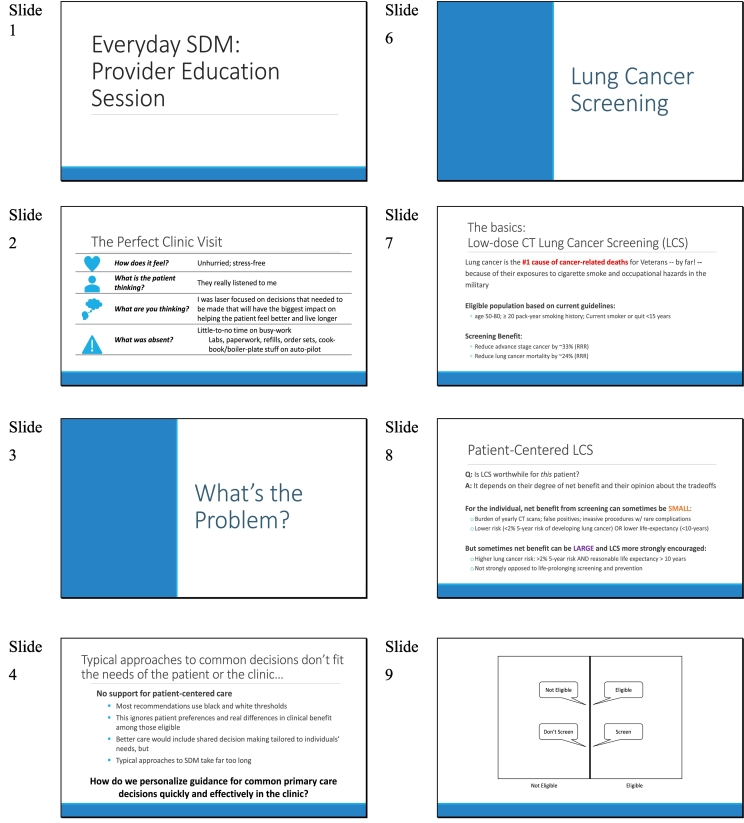

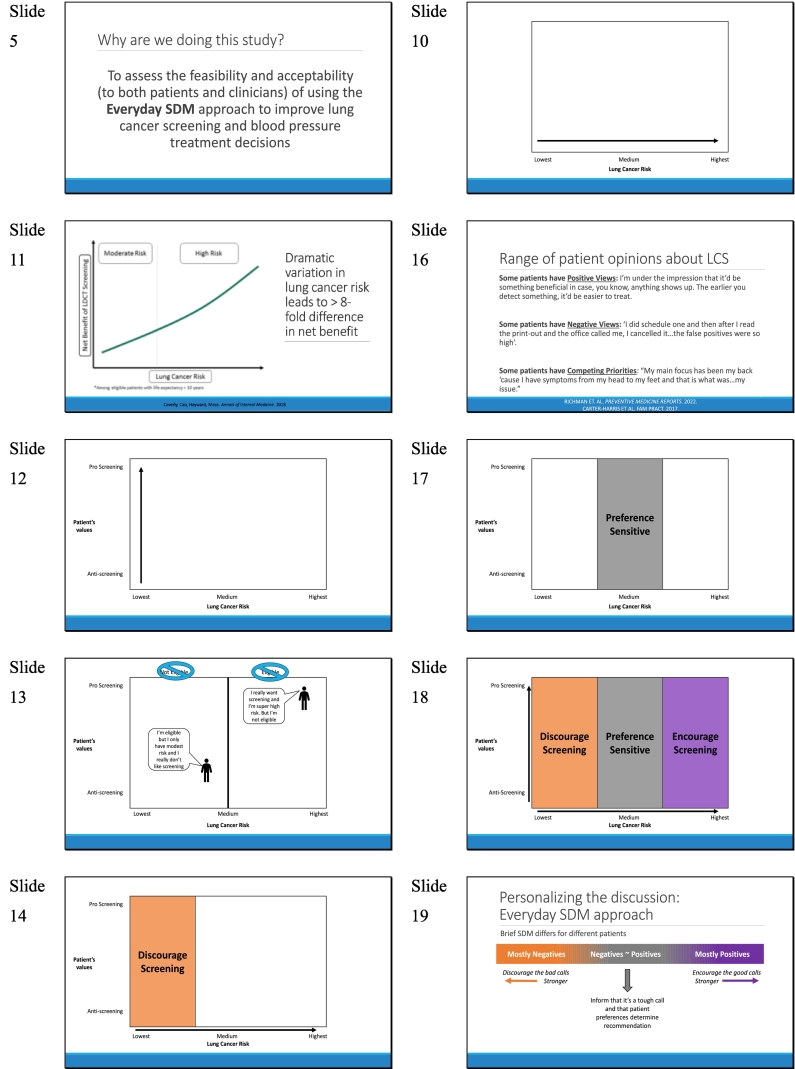

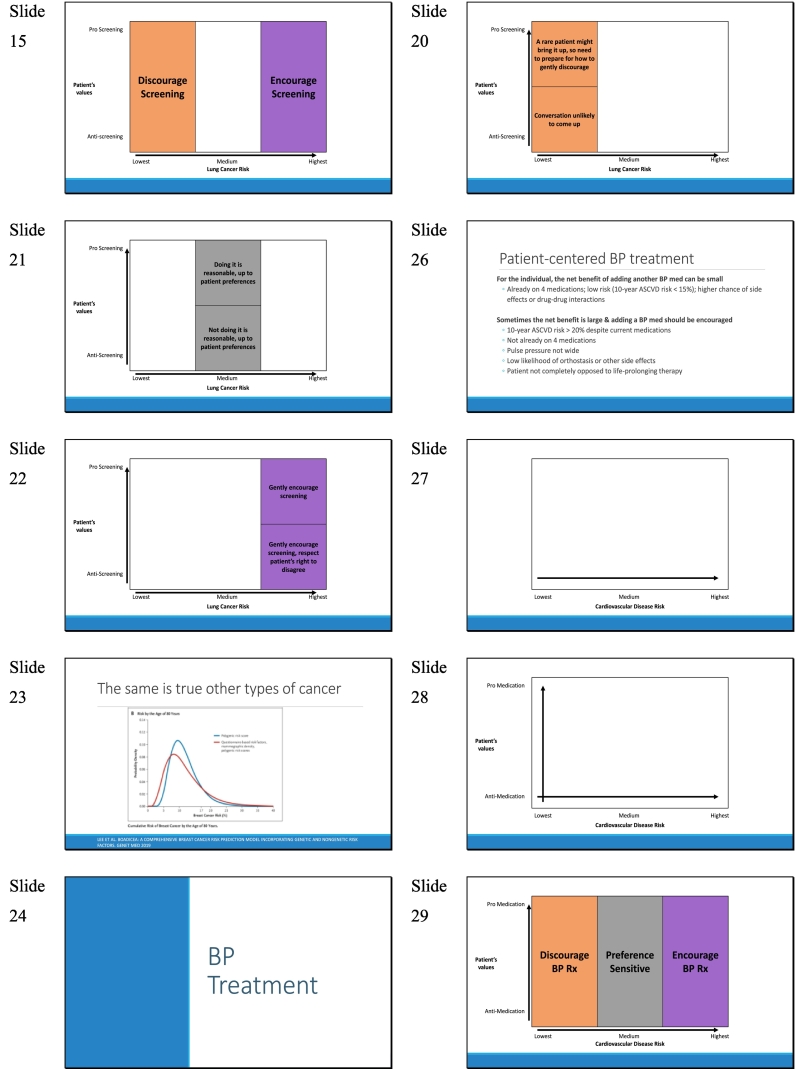

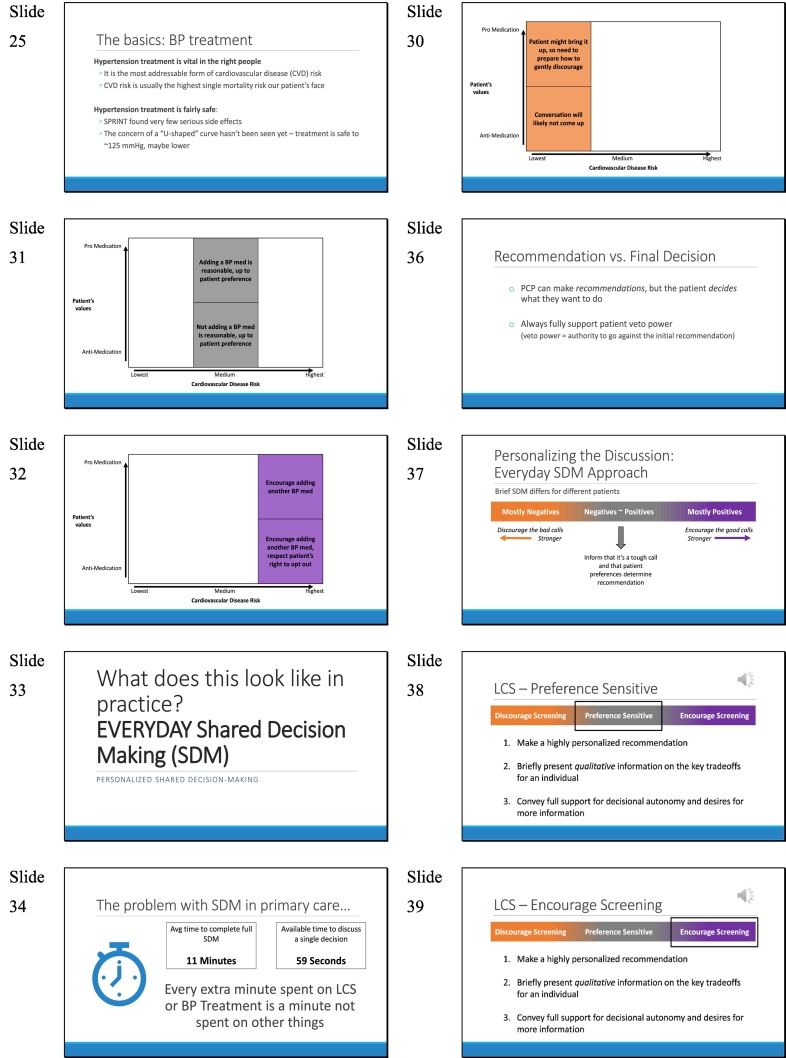

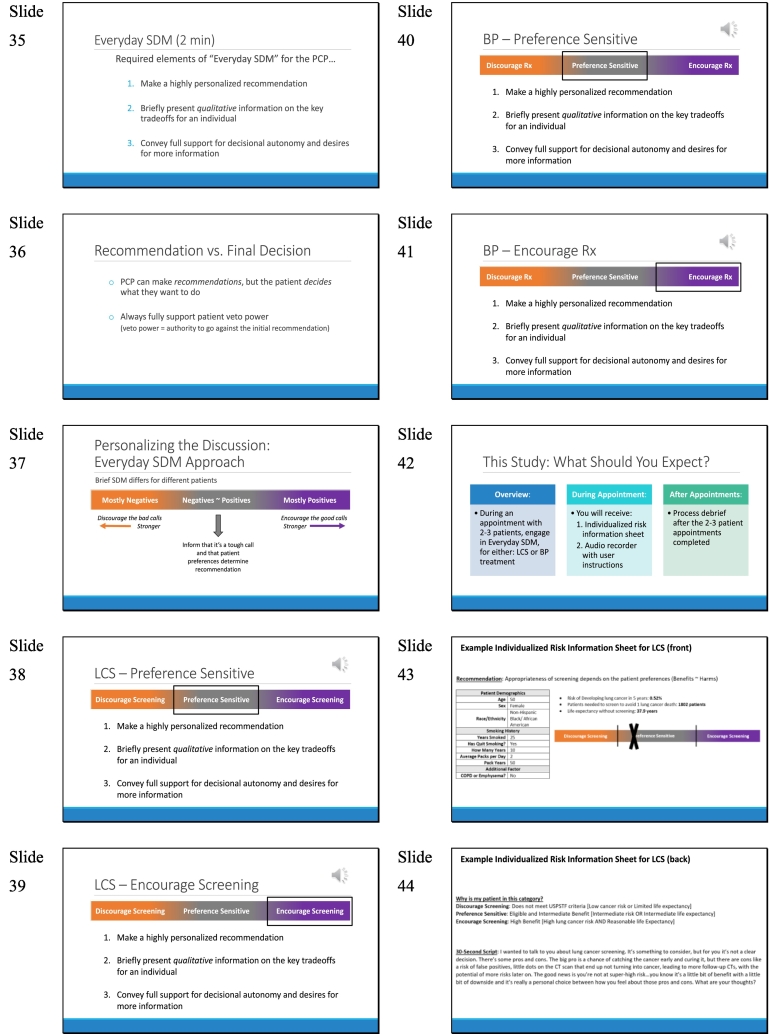

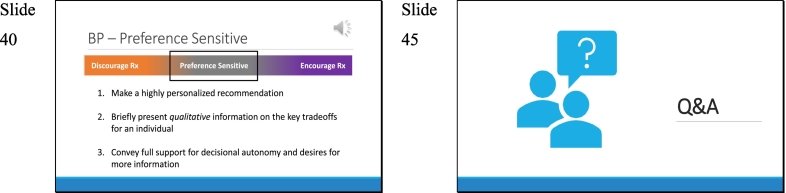


**Table:** 

To ensure reliability and face validity, the ZIP Scale was tested and refined by the team until agreement and consistency between team members was met. One specific revision was to item 2, which was separated into two subcategories (Item 2 A and 2B) based on the patient's recommendation zone. The scoring for Item 2 A, for patients categorized in the Encourage Zone, was based on the nuances of an Encourage zone recommendation. Specifically, in order to receive a “Fully completed” score for an encourage recommendation a single encourage recommendation must be made. Conversely, for Item 2B, for Preference-sensitive zone patients, to receive a “Fully completed” at least two treatment options must have been presented to the patient. For example, a provider could offer the option to intensify a BP medication or continue to take BP readings at home to gather more data. An additional change was to Item 5, “Did the provider support the patient as the final decision maker?”. It was clarified that in order to score a “Fully complete” the provider must express interest in the patient's thought process by asking a question to gauge preferences or by pausing to allow the patient time to gather their thoughts. During this refinement period, two items on the scale required further clarification: Item 2 (giving a recommendation) and Item 5 (supporting the patient as a final decision maker). Specifically, Item 2 was separated into Item 2 A and Item 2B, giving an encourage or preference sensitive recommendation, respectively. The scoring definitions were clarified so that for a medical encounter to receive “Full completed” score for an encourage recommendation a single encourage recommendation must be made (2 A), whereas to receive a “Fully completed” for a Preference Sensitive recommendation, at least two treatment options must have been presented to the patient. For example, a provider could offer the option to intensify a BP medication or continue to take BP readings at home to gather more data. Then, for Item 5, the definition was clarified to explicitly state what supporting the patient as a final decision-maker looked like. The new definition now includes that to score a “Fully complete” the provider must express interest in the patient's thought process by asking a question to gauge preferences or by pausing to allow the patient time to gather their thoughts.

**Appendix G:** Preference Sensitive ZIP Presentation Examples.

Initial Presentation (Min)	Physician Recommendation
1:20	“So basically, the blood pressure control. As you can imagine, right, each person's body is a little bit different. Some people you know may benefit from a more stricter blood pressure control than other people. We have this way of checking what your risk of heart disease and stroke is over a 10-year period. It's a calculator we use and (it isn't) a perfect thing, but it estimates your risk of having a heart attack or a stroke. But we know that good blood pressure control can reduce the risk of heart disease or stroke. OK? So that being said, your 10-year risk is about 14%. You know to have a heart attack or a stroke based on this calculator, your blood pressure is pretty well controlled, but it's still above 130 / 80 and that's kind of the goal. You are like borderline 131 / 81. The question we have is it worthwhile to kind of lower it a little bit further… because … (of) the risk factors you have and your risk of heart disease or stroke… maybe a little bit stricter control would be more beneficial for you, right?”
1:20	“For your blood pressure, we're going to go through kind of the script that they have for me. Because of your age, gender, race and blood pressure and cholesterol and all of these things, the chances of you having a heart attack in the next 10 years is about 9%. OK? And so, you're on a good cholesterol medication, you don't have diabetes, and you're on semaglutide to lose weight, which is a diabetes medication. You are on good blood pressure medications, and your blood pressure today was 129 / 81. You know, in the past it's been, you know, a little bit higher (than the) 30s. So, the question is would another medication lower your blood pressure and decrease your chances of having a heart attack? This is an individual decision that many people want, and many people may not want…”
3:07	PCP: How (was) your blood pressure … checks yesterday? Patient: … I think it was like 110/60 something and then the next three it was like 125/75. So, all within normal range. PCP Are you checking it at home? Patient I take it occasionally at home… PCP And what numbers do you get there? Patient Well, it can vary. You know, it can be like in the (111–119 range) to the 120 s. PCP: Got it. Patient: It's never high. PCP: Got it. Yeah, alright. Patient: Yeah. And I wanted to ask you, and I probably should have done this before. Metoprolol … besides… controlling my heart rate, it is a blood pressure med too? PCP: It can lower blood pressure a little bit. It's not like a dramatic lowering of blood pressure, but it can lower it a little bit. Patient: OK PCP: If we needed more blood pressure medications there's one called Carvedilol which lowers blood pressure more that we could switch you to. Or we can add a second medication. Patient: But are you pretty satisfied with what the readings are with what I'm taking? PCP. So, if they're like 120 s at home. Yeah, absolutely. Absolutely. Patient: OK PCP: You know, it says here that your … risk of having a heart attack over the next 10 years is like 18%/17.5%, and so the things that go into that are your age. We (have) no control over that: Race, gender, we can't change that. Patient: Right. PCP: Your cholesterol. We can look at that to see if there are any things we can intensify there. You don't have diabetes. You're not a smoker. Patient: right PCP: So you know the question of whether… you take a blood pressure medication (or) another medication is up to you. You're pretty good right now. Because … on this reading you are over 137. They, you know, are saying… maybe you could be a little bit lower around 120, but you're telling me you're 120 s at home, one teens. So, you know, one reading shouldn't drive (one) data point shouldn't drive this decision, but multiple ones. So that's what you're getting when you're the standardized patient, that's what you're getting at home, I don't think you really need to add one (medication). If you wanted to you could. 2080 OK.
2:25	“OK, so part of the discussion today is to talk about cardiovascular risk. That's something that I… So that's something that I am checking for, blood pressure, as well as cholesterol. There are ways that we can try gauge risk. Cardiovascular risk - Risk of heart attack or stroke. Yeah, particularly in the next 10 years or so. Part of this is whether we should add an additional, add a blood pressure medication to help reduce your risk for cardiovascular events. And you know, obviously, like all medications and things that we do, it's the individual's decision. And right now, your blood pressure is very well controlled. I mean it … sounds like you been getting at home. It's well controlled. And you know if you were to get it a little bit lower than that also would lower your risk for cardiovascular events. So you know, if you, if you're the type of person that doesn't mind taking other medication, that will continue to lower your risk for cardiovascular event, like I said, strokes, heart attacks… that is a good thing for you and for if you want to try not having an additional medication then you can you continue to evaluate and watch in the future?”
2:05	“And then so that kind of brings us to talking about your blood pressure, right. Today I see that the blood pressure was 140 / 77, which is a little bit higher than we normally like it…. So, I think we've checked it at home, it's been in like a better range at home, do you still have a machine at home? … I think what I would recommend is to keep checking it at home just to make sure that it's not starting to creep up on us, that it is closer to normal at home, and then I'll have one of the nurses give you a call to follow up in a week or two about how that blood pressure is going at home. The question then is the decision that we have to make is if you are a little bit higher… there is still probably some benefit to helping reduce your risk of having a heart attack and stroke by using more of the blood pressure medication… In that case, if you were still a little high, like in the 130 s, there … would be some benefit… with preventing a heart attack by using a higher dose of a lisinopril. OK, though the downside is making your blood pressure lower can sometimes come with side effects of like headedness or dizziness… so say we call in a week or two and you're still in the 130 s. (A)re you kind of in the mindset that I'd actually, I'd like to try and get that blood pressure a little bit lower or you know, I'm OK with where it is, right now?”
1:15	“And then in terms of blood pressure, you're kind of in this, you know, gray zone in terms of… you know, do we want to be more aggressive about it? Do we not need to be more aggressive about it? You know, if you're consistently above 140 and 90… you know 140 for top (number and) 90 for the diastolic number, the lower number, then usually we are encouraging you to either add some medication, you know really kind of double down on watching sodium and sugar, those things can affect your blood pressure. You're more in a gray zone where it's not perfect and I would love to see your blood pressure like in the 120 s and you know, 70s diastolic. But it's, yeah, we could go either way sort of. And so, for some people, you know, they really, you know, kind of want to cross (their) TS and dot (their) Is and be aggressive about it. And we can, you know… talk about adding a medication right now. You're on actually I don't know that we would even need to add anything. You're on lisinopril… but you're not at a max dose. You know you're on like a low … to medium dose. So, we could increase that.”
0:50	“There are a few things we should talk about. One of them, the thing that we should definitely talk about… is… your blood pressure, the goal for blood pressure is 1 less than 130 / 80. OK? That's the goal for it. Now, in some people, your blood pressure is usually in 130 s, and you know that may be OK for some people, but for somebody like you, you know your risk of heart attack in the next 10 years is about 11.5% or maybe higher given your history of smoking. OK, so… you may be somebody that is you know who their blood pressure is a little bit… better controlled (and) … who benefits from it. And one of the options is to just increase lisinopril from 5 to 10.”

**Appendix H:** Qualitative Research Reporting Guidelines (SRQR).

	Item Description	Location (or reason for not reporting)
Title & Abstract		
Title	Describe the nature and topic of the study. Identify the study as qualitative or indicate the approach or data collection methods.	Title Page
Abstract	Summarize the key elements of the study using the abstract format of the intended publication.	Abstract
Introduction		
Problem Formulation	Describe the problem/phenomenon studied, its significance, relevant theory and empirical work, and gaps in current knowledge.	Introduction; Paragraph 1
Purpose or research question	Describe the purpose of the study and specific objectives or questions.	Introduction; Paragraph 2
Methods		
Qualitative approach and research paradigm	Describe your qualitative approach, your guiding theory (if appropriate), and research paradigm, and reasons for your choices.	Methods; Paragraph 1
Researcher characteristics and reflexivity	Describe how researchers' characteristics may influence the research, including personal attributes, qualifications/experience, relationship with participants, assumptions, and/or presuppositions; potential or actual interaction between researchers' characteristics and the research questions, approach, methods, results and/or transferability.	Methods; Intervention
Context	Describe the setting/site(s) in which the study was conducted, why it was selected, and any other salient contextual factors that may influence the study.	Methods; PCP Recruitment and Education
Sampling strategy	Describe how and why research participants, documents, or events were selected; criteria for deciding when no further sampling was necessary, and the rationale for those criteria.	Methods; PCP Recruitment and Education
Ethical issues pertaining to human subjects	Describe any approval by an appropriate ethics review board and participant consent, or explain any lack thereof. Describe any other confidentiality and data security issues.	Methods; Ethical Considerations
Data collection methods	Describe the types of data collected; details of data collection procedures including (as appropriate) start and stop dates of data collection and analysis, iterative process, triangulation of sources/methods, and modification of procedures in response to evolving study findings. Describe your rationale for these choices.	Methods; Intervention
Data collection instruments and technologies	Describe any instruments (e.g., interview guides, questionnaires) and devices (e.g., audio recorders) used for data collection; describe if/how the instrument(s) changed over the course of the study.	Methods; Intervention
Units of study	Describe the number and relevant characteristics of participants, documents, or events included in the study. Describe the level of participation.	Results
Data processing	Describe the methods for processing data prior to and during analysis, including transcription, data entry, data management and security, verification of data integrity, data coding, and anonymisation / deidentification of excerpts.	Methods; Acceptability
Data analysis	Describe the process by which inferences, themes, etc. were identified and developed, including the researchers involved in data analysis; usually references a specific paradigm or approach. Describe why you chose this process.	Methods; Acceptability
Techniques to enhance trustworthiness	Describe any techniques to enhance trustworthiness and credibility of data analysis,(e.g., member checking, triangulation, audit trail). Describe why you chose these techniques.	Methods; Acceptability
Results		
Synthesis and interpretation	Describe the main findings (e.g., interpretations, inferences, and themes); might include development of a theory or model, or integration with prior research or theory.	Results; All paragraphs
Links to empirical data	Provide evidence (e.g., quotes, field notes, text excerpts, photographs) to substantiate analytic findings.	Results; Acceptability - Interviews
Discussion		
Integration with prior work, implications, transferability, and contribution(s) to the field	Summarize the main findings, explain how findings and conclusions connect to, support, elaborate on, or challenge conclusions of earlier scholarship; discuss the scope of application/generalizability; identify unique contribution(s) to scholarship in a discipline or field.	Discussion; Paragraph 1–3
Limitations	Discuss the trustworthiness and limitations of findings	Limitations
Other		
Conflicts of interest	Describe any potential sources of influence or perceived influence on study conduct and conclusions. Describe how these were managed.	Acknowledgements; Conflicts of Interest Summary
Funding	Describe sources of funding and other support. Describe the role of funders in data collection, interpretation, and reporting.	Acknowledgements; Paragraph 2

## Appendix B ZIP Scale Item and Composite Scores by Recommendation Zone and Cohort

0 = Did not Attempt, 1 = Partially Complete, 2 = Fully Completed.

a = Lung Cancer Screening.

b = Blood Pressure treatment.

c = Encourage Zone.

d = Preference-Sensitive Zone.

Item 1: Personalized Recommendation. Did the PCC communicate that the recommendation was personalized to the patient's specific risk factors?

Item 2 A: If the patient is in the encourage zone, did the PCC give an ‘encourage’ recommendation? Item 2B: If the patient is in the ‘preference-sensitive’ zone, did the PCC give a ‘preference-sensitive’ recommendation?

Item 3: Did the PCC offer qualitative information on pros and cons?

Item 4: Did the PCC support an explicit patient request for more information?

Item 5: Did the PCC support the patient as the final decision maker?

*Patients with no ZIP Score (i.e., no medical encounter recording, missing survey) have been deleted from this table.

## Appendix E Blood Pressure Patient Semi-Structured Interview Guide

Veteran Interview Guide.

Introduction

Thank you for completing the survey, I just have a few follow-up questions I wanted to discuss with you. This interview should only take about 10–15 min. Our goal is just to understand more about your thought process with decision making, perspectives on benefits and harms, and interactions with your provider. As we have mentioned before, all of this will remain completely confidential and will not get back to your provider. Therefore, feel free to answer openly and honestly. But you are free to not answer any questions you would not like to.

As I mentioned, I will be recording this, so we have an accurate record of what you tell us. If you would like me to pause or turn off the recorder at any time, please let me know.

*Turn recorder on*.

This is participant # _____.

ZIP Approach Debrief.

1. During your appointment did you doctor discuss BP treatment options with you?
  a. If so,
    i. How did the conversation go?
  b. If no – Skip to Decision- Making questions
2. Did you have any questions about BP treatment for your doctor after the conversation?
  a. Do you have any questions that you didn't ask your doctor that you wish you would have?
3. How strongly did you feel your provider either recommended or didn't recommend BP treatment?
  a. Tell me more about why you felt that?
4. We have been training some providers in a new communication technique to include more personalization in their recommendations, do you feel the conversation was personalized to you?
  a. Was this reflected in your conversation?
5. Would you like to see this personalized communication approach used more frequently with other preventive care topics?

Decision Making.

6. I see here on the survey you answered (Q15 about control with medical decisions) Will you please tell me more about how you like to make medical decisions? Why?
  a. Does your control over decision making change based on the health topic or health concern?

7. At this time have you made a decision about BP treatment at this time?
  a. Why did you make this decision?

Benefits/Harms.

8. What do you think are potential benefits of starting BP treatment?
  a. What do you think is the most important Benefit? Why?
9. What do you think are potential harms of starting BP treatment?
  a. How worried are you about experiencing a harm? Why?
10. In your opinion, how do you think the risks compare to the benefits?

11. How worried are you about heart attacks and strokes?
  a. In your opinion, how helpful is adding a BP medication?

Conclusion

That is all of the questions I wanted to ask you. Do you have anything else you would like to add? Do you have any questions for me?

*Turn recorder off*.

Thank you very much for participating in this survey and interview. We greatly appreciate your time and thoughtful input. Here is your $25 gift card as a thank you.

## Appendix F Physician Semi-Structured Interview Guide

Provider Interview Guide.

Introduction

Thank you for your participation in the pilot study. Now that you have been using the ZIP Approach ‘Zeroing in on Individualized, Patient-Centered Decisions’ within in the clinic for a little bit now, we just wanted to follow-up and see how things are going. The goal of the conversation is to understand more about your thought process with decision making, perspectives on benefits and harms, and interactions with your provider. As we have mentioned before, all of this will remain completely confidential. Therefore, feel free to answer openly and honestly. But also you don't need to answer anything you don't want to.

As I mentioned, I will be recording this, so we have an accurate record of what you tell us. If you would like me to pause or turn off the recorder at any time, please let me know.

*Turn recorder on*.

This is participant #______.

ZIP Approach Debrief.

1. First, please start by telling me how using the ZIP approach in the clinic went?
  a. What was your process?
  b. What was easy?
  c. What was difficult?
  d. What barriers did you run into?
2. How strictly did you stick to the scripts given? Why?
  a. What about the scripts worked?
  b. What didn't work?
3. How long did it take to complete the ZIP approach?
4. How effective is this approach at accomplishing shared decision making?
  a. Was it missing anything?
5. In your opinion, how receptive were your patients to the conversation?
  a. Did you notice any different in their reactions towards either LCS or BP treatment?
6. Outside of the [1], [2], [3] patients we gave you information about, did you use this approach with any other patients?
  a. If so, how did it go?
  b. How did you determine the recommendation group?
7. Moving forward, do you see yourself continuing to incorporate this type of communication strategy into your practice?

8. Would you like to see this personalized communication approach used more frequently with other preventive care topics?

Benefits/Harms of LCS.

I now want to ask you first about some of your perceptions on the benefits and harms of lung cancer screening specially:

9. What do you think is the most important or significant benefit of lung cancer screening? Why?
10. How worried are you about your patients dying from lung cancer without screening? Why?
11. What do you think is the most important or significant harm of lung cancer screening? Why?
12. What is your perception of the balance of risks and benefits for lung cancer screening?
13. Did any of the perception about risks and benefits change after using the ZIP approach?

Benefits/Harms of BP Treatment.

Next I want to ask about some of your perceptions on the benefits and harms of Blood pressure treatment:

14. What do you think is the most important or significant benefit of [Starting/Stopping] Blood pressure medications? Why?
15. How worried are you about your patients having a heart attack or stroke before [Starting/Stopping] Blood pressure treatment? Why?
16. What do you think is the most important or significant harm Blood Pressure Treatment Medication? Why?
17. What is your perception of the balance of risks and benefits for Blood pressure treatment?
18. Did any of the perception about risks and benefits change after using the ZIP approach?

Wrap-up.

That is all of the questions I wanted to ask you. Do you have anything else you would like to add? Do you have any questions for me?

Thank you very much for participating in this pilot project and this final interview. We greatly appreciate all of your time and thoughtful input and consideration.
